# Eating disorders. What about males?

**DOI:** 10.1192/j.eurpsy.2023.1801

**Published:** 2023-07-19

**Authors:** G. Strada Herrera, C. Pérez Sobrino, M. Díaz Marsá

**Affiliations:** Hospital Clínico San Carlos, Madrid

## Abstract

**Introduction:**

Eating disorders (ED) historically been adressed as illnesses that only affect young adolescent females. ED’s in males have been documented in literature as early as the 1960’s; yet men continue to be under represented on research on the topic. For decades, the Diagnostic and Statistical Manual of Mental Disorders (DSM) perpetuated the invisibility of males by including amenorrhea as a diagnostic criterion. It was not until 2013 that male inclusion was endorsed thorught the removal of that criterion. It is estimated that one in four people affected with and ED is male.

It is estimated that one in four people affected with and ED is male. The proportion of males reporting lifetime prevalence of Binge eating disorder (BED) was far greater than for Anorexia nervosa (AN) or Bulimia nervosa (BN); the female versus male ratio of BED prevalence was 3:1. AN is the most life-threatening ED, but is least frequently seen in male populations; researchers suggest this is because most men are not interested in the emaciated, thin look.

**Objectives:**

This poster aims to recognize the presence of ED’s in males and raise awareness on this topic.

**Methods:**

Case report and literature review

**Results:**

We present the case of a 50-year-old man with long-standing AN, who had never undergone mental health follow-up. He is referred to psychiatrist by his primary care provider (PCP) due to depressive symptoms. His medical history included vitamine D insufficiency and osteoporosis. At the age of 19 he was obese (BMI 35) and from the age of 23 he started to present dietary restriction after a social event. He had never self-induced vomiting, use of laxatives, binge eating or compulsive exercise. He reported no history or current substance use disorder. BMI at first consultation was 17,6 and showed fear of weight gain. Antidepressant therapy was started and patient was referred to a specialized therapist, nutritionistand nurse.

**Conclusions:**

Overall, the findings demand clinicians develop awareness about ED in males to advance illness management and enhance long-term prognosis. In our case, the delay in receiving treatment has probably led to greater morbidity and chronicity. PCP’s play a key role in detection of ED’s as the often act as a first point of contact for men accesing the health care system. While assesing and ED, the PCP should include general questions on eating habits in their intake interview. Once an a ED is suspected, the first few minutes of the encounter are crucial to gain trust and buy-in from the patient. Once buy-in from the patient is gained, a complete physical exam and diagnostic work-up is required. Priority referrals to the following professionals are critical: psychiatrist, therapist, dietician or nutritionist, and ED specialist if available.

**Disclosure of Interest:**

None Declared

